# Coumarin–Dithiocarbamate Derivatives as Biological Agents

**DOI:** 10.3390/ijms26199667

**Published:** 2025-10-03

**Authors:** Piotr Wiliński, Aleksander Kurzątkowski, Kinga Ostrowska

**Affiliations:** Department of Organic and Physical Chemistry, Faculty of Pharmacy, Medical University of Warsaw, Banacha 1, 02-097 Warsaw, Poland; s091877@student.wum.edu.pl (P.W.); s091796@student.wum.edu.pl (A.K.)

**Keywords:** coumarin derivatives, dithiocarbamate, biological activity

## Abstract

Coumarin derivatives, whether natural or synthetic, have attracted considerable interest from medicinal chemists due to their versatile biological properties. Their appealing pharmacological activities—such as anticancer, anti-inflammatory, neuroprotective, anticoagulant, and antioxidant effects—combined with the ease of their synthesis and the ability to introduce chemical modifications at multiple positions have made them a widely explored class of compounds. In the scientific literature, there are many examples. On the other hand, dithiocarbamates, originally employed as pesticides and fungicides in agriculture, have recently emerged as potential therapeutic agents for the treatment of serious diseases such as cancer and microbial infections. Moreover, dithiocarbamates bearing diverse organic functionalities have demonstrated significant antifungal properties against resistant phytopathogenic fungi, presenting a promising approach to combat the growing global issue of fungal resistance. Dithiocarbamates linked to coumarin derivatives have been shown to exhibit cytotoxic activity against various human cancer cell lines, including MGC-803 (gastric), MCF-7 (breast), PC-3 (prostate), EC-109 (esophageal), H460 (non-small cell lung), HCCLM-7 (hepatocellular carcinoma), HeLa (cervical carcinoma), MDA-MB-435S (mammary adenocarcinoma), SW480 (colon carcinoma), and Hep-2 (laryngeal carcinoma). Numerous studies have revealed that the inclusion of a dithiocarbamate moiety can provide central nervous system (CNS) activity, particularly through inhibitory potency and selectivity toward acetylcholinesterase (AChE) and monoamine oxidases (MAO-A and MAO-B). Recently, it has been reported that coumarin–dithiocarbamate derivatives exhibit α-glucosidase inhibitory effects and also possess promising antimicrobial activity. This study presents an overview of recent progress in the chemistry of coumarin–dithiocarbamate derivatives, with a focus on their biological activity. Previous review papers focused on coumarin derivatives as multitarget compounds for neurodegenerative diseases and described various types of compounds, with dithiocarbamate derivatives representing only a small part of them. Our work deals exclusively with coumarin dithiocarbamates and their biological activity.

## 1. Introduction

**Figure 1:**
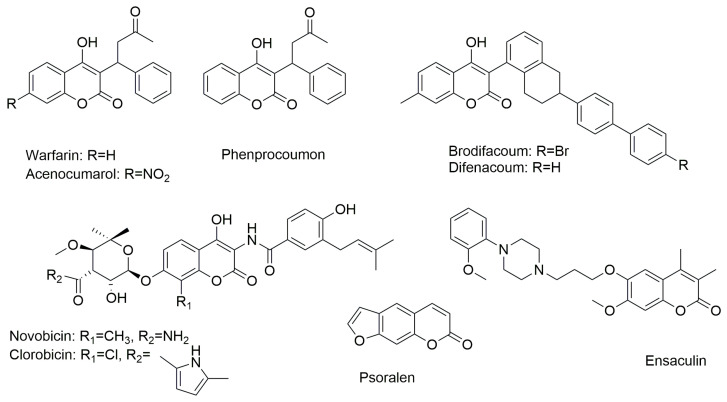
Chemical structure of coumarin-based drugs.

Coumarins, chemically known as 1,2-benzopyrones (benzene ring fused to an α-pyrone), are a group of secondary metabolites primarily found in plants, as well as in fungi and some microorganisms. Their synthesis has gained significant interest due to their broad application in pharmaceuticals, food additives, cosmetics, and perfumes. Additionally, coumarins are widely used as fluorophores in the labeling of biomolecules and for detecting pH, metal ions, and microenvironment polarity [1]. Natural and/or synthetic coumarin derivatives have attracted extensive attention from medicinal chemists due to their structural versatility, ease of synthesis, and the possibility for chemical modifications at multiple positions. Most importantly, these compounds are of great interest because of their diverse and significant biological activities. Coumarin’s straightforward molecular framework allows it to dissolve well in many organic solvents and exhibit good bioavailability. Due to its ability to easily combine with different chemical groups, it is frequently used as a core scaffold in the development of new pharmaceutical compounds [2]. Several clinically approved drugs, including warfarin, novobiocin, and brodifacoum, incorporate coumarin as a key component of their chemical structure. 4-Hydroxycoumarin derivatives, such as warfarin, acenocoumarol, phenprocoumon, as well as brodifacoum and difenacoum, are used as vitamin K antagonists and serve as first-line oral anticoagulants [3,4,5] (Figure 1). Naturally occurring aminocoumarins, such as novobiocin and clorobiocin, exhibit a broad spectrum of activity as antibiotics effective against Gram-positive bacteria, including methicillin-resistant bacterial strains [6] (Figure 1). Psoralen and its 8- or 5-methoxy derivatives are used in a variety of skin diseases and cutaneous T-cell lymphomas [7] (Figure 1). Ensaculine, on the other hand, acts as an AChE (acetylcholinesterase) inhibitor [6,7] (Figure 1).

The pharmacological profiles of various natural and/or synthetic coumarin derivatives are very broad and have been described in numerous scientific publications. The literature includes coumarins with antiviral, anti-cancer, antibacterial, anti-inflammatory, antioxidant, anti-tuberculosis activities, and many others [8,9].

**Figure 2:**
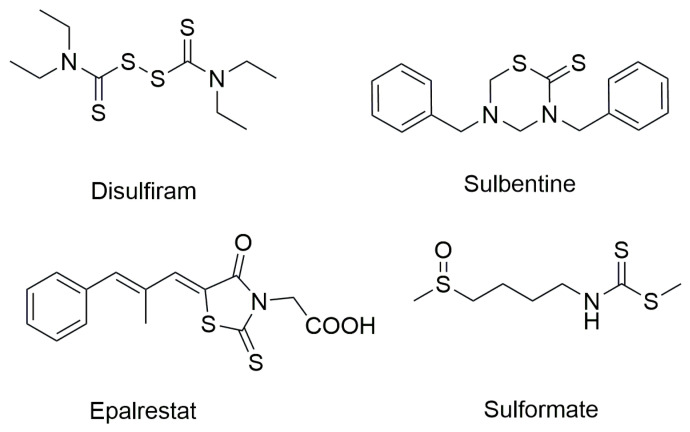
Chemical structure of dithiocarbamate-based drugs.

On the other hand, organic dithiocarbamates, recognized as versatile scaffolds in organic chemistry, have found applications across numerous fields, including organic synthesis, environmental and material sciences, the polymer industry, agrochemicals, and pharmaceuticals [10,11,12,13,14,15,16,17,18,19]. Notably, they also demonstrate promising biological activities such as antibacterial, antioxidant, anticancer, and antifungal effects. Originally employed as pesticides throughout the 20th century, dithiocarbamates are among the simplest organosulfur compounds and are widely acknowledged for their metal-chelating abilities. However, their chemical and pharmacological potential extends far beyond metal binding. Over the last decade, extensive studies have revealed a broad spectrum of chemical and medicinal properties associated with dithiocarbamate derivatives [20,21,22,23,24,25]. The dithiocarbamate group forms the core structure of Disulfiram, a drug originally used for treating chronic alcoholism, which has more recently shown potential as an anticancer agent [26,27]. Additionally, the dithiocarbamate moiety serves as a key pharmacophore in other marketed drugs, including sulbentine, epalrestat, and sulforamate, which exhibit a range of biological activities [28,29,30] (Figure 2).

The combination of a dithiocarbamate moiety with an active pharmacophore results in an improved biological profile of the final compound. For this reason, the synthesis of their derivatives with coumarins appears to be a purposeful approach. So far, the literature has revealed the potential of coumarin–dithiocarbamate hybrids as compounds acting on the central nervous system, cytotoxic agents, derivatives with antimicrobial activity, and α-glucosidase inhibitors. This is a relatively new topic, but undoubtedly worth summarizing. Therefore, in this work, we have undertaken the task of collecting and reviewing the current findings and achievements related to this subject.

## 2. Biological Effects of Coumarins Containing the Dithiocarbamate Moiety

### 2.1. Coumarin–Dithiocarbamate Derivatives with Cytotoxic Activity

**Figure 3:**
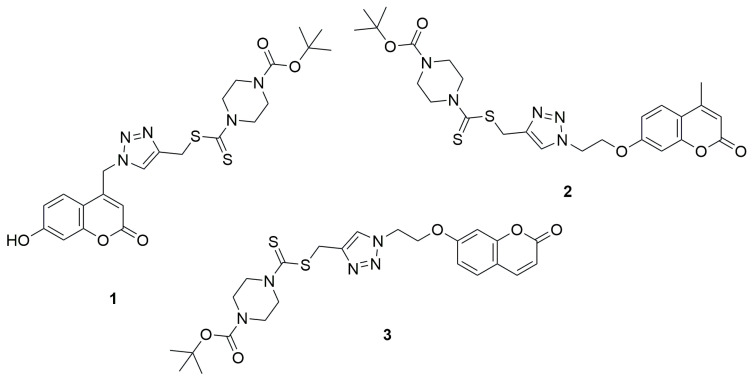
Coumarin derivatives synthesized by Duan’s group [31].

The first literature reports on coumarin–dithiocarbamate hybrids with cytotoxic activity date back to 2013. Duan et al. demonstrated that 1,2,3-triazole–dithiocarbamate hybrids containing a coumarin moiety exhibited potent anticancer activity [31]. The autors evaluated the inhibitory concentrations (IC_50_), representing the amount of compound needed to suppress 50% of tumor cell growth, against four human cancer cell lines: MGC-803 (gastric), MCF-7 (breast), PC-3 (prostate), and EC-109 (esophageal). The measurements were carried out using the MTT assay technique. Among the tested compounds containing a coumarin moiety in their structure, *tert*-butyl 4-(((1-((7-hydroxy-2-oxo-2H-chromen-4-yl)methyl)-1H-1,2,3-triazol-4-yl)methylthio)carbonothioyl)piperazine-1-carboxylate (**1**) exhibited good anticancer activity against MGC-803 (IC_50_ = 4.96 ± 0.78 µM) and MCF-7 (IC_50_ = 10.44 ± 2.34 µM) cell lines in comparison to the reference compound 5-fluorouracil (5-FU) (IC_50_ = 7.01 ± 1.34 µM and 7.54 ± 0.7 µM, respectively). Other tested coumarin derivatives were less active, MGC-803 (IC_50_ = 76.90 ± 3.56 µM) and MCF-7 (IC_50_ = 90.38 ± 4.05µM) for *tert*-butyl-4-(((1-(2-(4-methyl-2-oxo-2H-chromen-7-yloxy)ethyl)-1H-1,2,3-triazol-4-yl)-methylthio)carbonothioyl)piperazine-1-carboxylate (**2**) and for *tert*-butyl-4-(((1-(2-(2-oxo-2H-chromen-7-yloxy)ethyl)-1H-1,2,3-triazol-4-yl)methylthio)-carbonothioyl)piperazine-1-carboxylate (**3**) IC_50_ = 67.05 ± 2.98 µM and 79.80 ± 3.09 µM, respectively (Figure 3).

**Figure 4:**
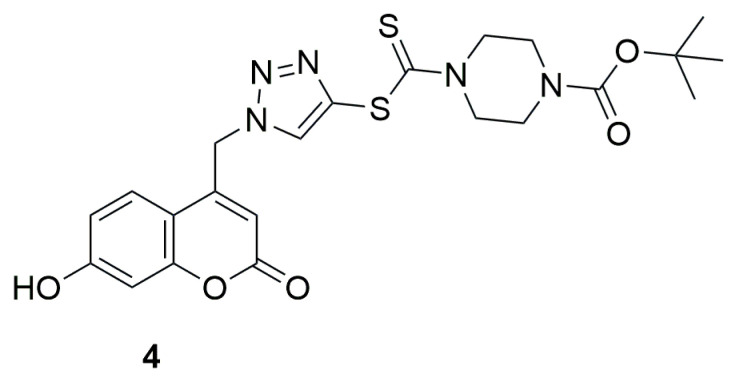
Structure of coumarin derivative 4-(((1-((7-hydroxy-2-oxo-2H-chromen-4-yl)methyl)-1H-1,2,3-triazol-4-yl)thio)carbonothioyl)piperazine-1-carboxylate (**4**).

In 2014, Ye and collaborators investigated a series of coumarin-1,2,3-triazole-dithiocarbamate hybrids as potent lysine specific demethylase 1 (LSD1) inhibitors, as it has been proven that downregulation of its expression or inhibition of its activity can suppress cancer progression [32]. Among the tested compounds *tert*-butyl 4-(((1-((7-hydroxy-2-oxo-2H-chromen-4-yl)methyl)-1H-1,2,3-triazol-4-yl)thio)carbonothioyl)piperazine-1-carboxylate (**4**) exhibited strong and reversible inhibition of lysine-specific demethylase 1 (LSD1), with an IC_50_ value of 0.39 ± 0.15 µM (Figure 4). The result for compound **4** was 74 times more better than the reference compound tranylcypromine (2-PCPA) (IC_50_ = 28.73 ± 1.21 µM). Further studies demonstrated that compound **4** was effective both at the recombinant protein level and in cells, where it increased the levels of histone (histone H3) methylation marks H3K4me1, H3K4me2, and H3K9me2.

**Figure 5:**
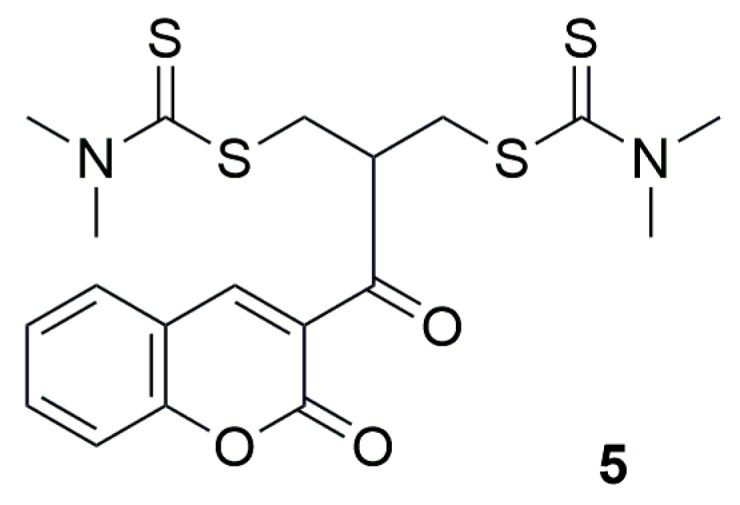
Structure of coumarin derivative 2-(2-oxo-2H-chromene-3-carbonyl)propane-1,3-diylbis(dimethylcarbamodithioate) (**5**).

The authors of the next work decided to synthesize a series of new derivatives incorporating various heterocyclic systems, including coumarin, thiazole ring, benzo[b]thiophene, quinoline or pyridine ring [33]. Among the synthesized compounds, 2-(2-oxo-2H-chromene-3-carbonyl)propane-1,3-diylbis(dimethylcarbamodithioate) (**5**) and 2-(quinoline-3-carbonyl)propane-1,3-diylbis(dimethylcarbamodithioate) exhibited excellent activity and represented promising drug-like scaffolds (Figure 5).

The compound **5** demonstrated activity against the human non-small cell lung cancer cell line H460, with IC_50_ value of 0.66 µM. In contrast, the compounds containing other heterocyclic moieties, such as pyrazolo [1,5-a]pyridine, imidazo [1,2-a]pyridine, 2-methylimidazo [1,2-a]pyridine, 1H-benzo[d]imidazole, or 1H-pyrrole, showed a significant decrease in activity, with some derivatives losing their cytotoxic effect entirely, from IC_50_ = 2.2 µM to 151.4 µM.

**Figure 6:**
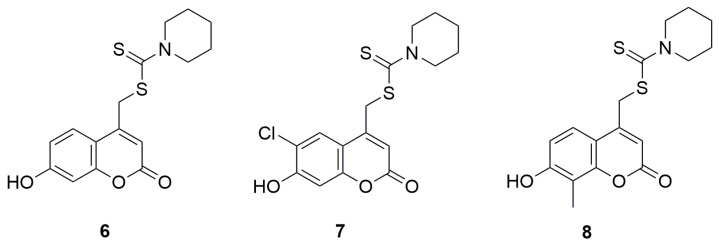
Coumarin derivatives synthesized by Haiyan’s group [34].

Another group of coumarin derivatives with anticancer activity, containing the dithiocarbamate fragment was evaluated by the methyl thiazolyl tetrazolium (MTT) method against hepatoma carcinoma cells HCCLM-7, cervical carcinoma cells Hela, mammary adenocarcinoma cells MDA-MB-435S, colon carcinoma cells SW-480, laryngocarcinoma cells Hep-2, and mammary adenocarcinoma cells MCF-7 [34]. Derivatives of 7-hydroxycoumarin, 6-chloro-7-hydroxycoumarin, and 8-methyl-7-hydroxycoumarin were investigated, each bearing a methylamine-1-carbodithioate moiety at the C-4 position of the coumarin ring and incorporating various aliphatic amines such as diethylamine, piperidine, morpholine or phenylpiperazine. The results showed that the best activity was observed for derivatives containing a piperidine moiety in their structure. The activity of (7-hydroxy-2-oxo-2H-chromen-4-yl)methylpiperidine-1-carbodithioate (**6**) (Figure 6) against all six tested cell lines was better that of 5-FU. Particularly low IC_50_ values for compound **6** were obtained against HCCLM-7 and MCF-7 cell lines, amounting to, respectively, 3.5 μM and 3.0 μM while for 5-FU it was 18.6 μM and 8.1 μM, respectively. Similar results were obtained for (6-chloro-7-hydroxy-2-oxo-2H-chromen-4-yl)methylpiperidine-1-carbodithioate (7), 12.3 μM against Hela, 11.6 μM against SW-480, and 44 μM against Hep-2, while it was 128.7 μM, 91 μM and 44 μM for 5-FU, respectively. Compound 8-(7-hydroxy-8-methyl-2-oxo-2H-chromen-4-yl)methyl piperidine-1-carbodithioate exhibited similar behavior, with IC_50_ values 2.5 μM against Hela, 15.3 μM against SW-480 and 7.8 μM against Hep-2. In summary, the introduction of an additional methyl group at the C-8 position of the coumarin ring or a chlorine atom at the C-6 position did not significantly affect the activity. However, the substitution of an additional hydroxyl group or the replacement of piperidine with another amine resulted in a drastic reduction of cytotoxic activity, which was significantly lower than that of the reference compound.

**Figure 7:**
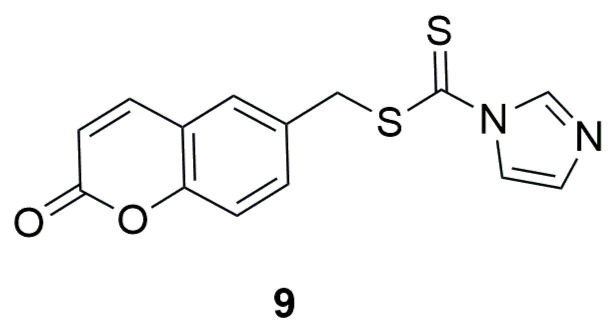
Structure of coumarin derivative (2-oxo-2H-chromen-6-yl)methyl-1H-imidazole-1-carbodithioate (**9**).

In 2021, the research group led by Zhu investigated novel coumarin–dithiocarbamate derivatives (IDs) as potential anti-colorectal cancer (CRC) agents [35]. The researchers focused on the modification of the C-6 position of the coumarin ring by introducing, as in the previous study, a methylamine-1-carbodithioate moiety at this position. To evaluate the in vitro anticancer activity of the compounds, three different human colorectal cancer cell lines—RKO, SW620, and SW480—were used, with 5-fluorouracil as a positive control. The best activity in the MTS assay was shown by (2-oxo-2H-chromen-6-yl)methyl 1H-imidazole-1-carbodithioate (**9**) (Figure 7), with cell viabilities of RKO, SW620, and SW480 lines at 29.43%, 43.94%, and 20.18%, respectively. In contrast, the viabilities of the same cell lines after treatment with 5-FU were 40.73%, 33.57%, and 43.87%, respectively.

The results of the MTS (3–(4,5-dimethylthiazol-2-yl)-5–(3-carboxymethoxyphenyl)-2–(4-sulfophenyl)-2H-tetrazolium) assay showed that replacing the imidazole moiety with aliphatic amines—including linear alkyl chains, four- to seven-membered aliphatic cyclic amines, morpholine, substituted piperazines, or piperidine—weakened the antiproliferative activity against these cell lines. Only for derivative **9**, the IC_50_ values were very promising and amounted to 6.398 μM for RKO, 8.809 μM for SW620, and 3.568 μM for SW480, respectively. The viability of NCM460 cells (normal human colon epithelial cells) after 48 h treatment with compound **9** and 5-FU at a concentration of 10 μM exceeded 50%, amounting to 80.84% for compound **9** and 80.36% for 5-FU. The IC_50_ value of (2-oxo-2H-chromen-6-yl)methyl-1H-imidazole-1-carbodithioate (**9**) against NCM460 cells was determined to be 23.89 μM. Additionally, the tested compound effectively inhibited the colony formation of SW620 and SW480 cells in a concentration-dependent manner (ranging from 0 to 32 μM). In an additional HTRF (Homogeneous Time-Resolved Fluorescence) assay, compound **9**, at a concentration of 10 μM, demonstrated potential inhibitory activity against BRD4 (bromodomain-containing protein 4), achieving 68.9% inhibition of the BD1 domain. For comparison, JQ1 (a thienotriazolodiazepine derivative and potent inhibitor of the BET family of bromodomain proteins, including BRD2, BRD3, and BRD4), used as a positive control, strongly inhibited both BRD4 (BD1) and BRD4 (BD2) under the same conditions, with inhibition rates exceeding 98%. The IC_50_ value of compound **9** against BRD4 (BD1) was determined to be 7.8 μM.

**Figure 8:**
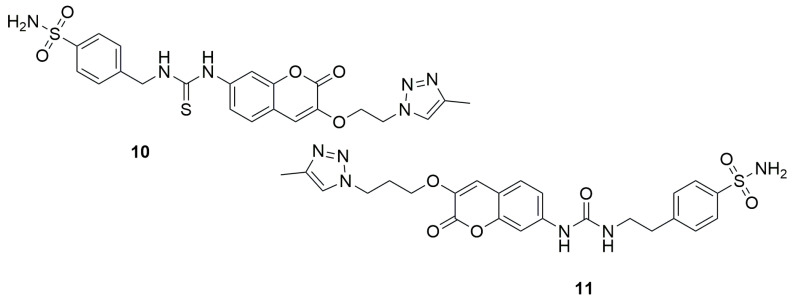
Coumarin derivatives synthesized by Begines’s group [36].

A slightly different set of coumarin structure modifications was carried out in 2024 by the research group led by Begines [36]. The authors synthesized a series of novel coumarin derivatives based on hybridization with arylsulfonamide or biotin scaffolds and tested them as inhibitors of four different human carbonic anhydrase isoforms: hCA I, II, IX, and XII, which are involved in the pathogenesis and progression of many types of solid tumors. *N*-[[(4-Aminosulfonyl)phenyl]methyl]-*N’*-[[(4-methyl-1*H*-1,2,3-triazol-1-yl)ethoxy]-2-oxo-2*H*-1-benzopyran-7-yl]thiourea (**10**) (Figure 8), a coumarin-sulfonamide compound containing a thiourea group and a triazole linker, exhibited the strongest inhibitory effect on hCA XII, with a KI value of 7.5 nM, and demonstrated excellent selectivity compared to hCA I. Meanwhile, N-[[(4-aminosulfonyl)phenyl]ethyl]-N’-[[(4-methyl-1H-1,2,3-triazol-1-yl)propoxy]-2-oxo-2H-1-benzopyran-7-yl]urea (**11**) turned out to be the most effective inhibitor of hCA IX, showing a KI of 6.3 nM—making it four times more potent than the reference drug acetazolamide (AAZ), which has a KI of 25 nM. Additionally, compound **11** displayed outstanding selectivity over the hCA I and II isoforms.

### 2.2. Coumarin–Dithiocarbamate Derivatives with Central Nervous System (CNS) Activity

**Figure 9:**
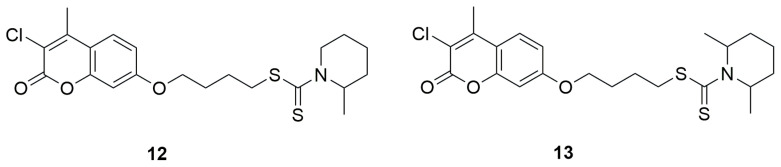
Coumarin derivatives synthesized by He’s group [37].

In 2018, a series of new coumarin derivatives incorporating a dithiocarbamate moiety was designed, synthesized, and tested as multitarget agents for the treatment of Alzheimer’s disease [37]. A series of derivatives with various substituents at the C-2 and C-3 positions of the coumarin ring was obtained. Subsequently, the effect of the linker length between the coumarin moiety and the dithiocarbamate group on biological activity was investigated, using linkers ranging from two to five carbon atoms in length. Considering both the inhibitory potency and selectivity toward acetylcholinesterase (AChE) and monoamine oxidase (MAO-A and MAO-B), the linker with four carbon atoms (m = 4) was identified as the optimal length for connecting the coumarin core with the dithiocarbamate group. In the final stage of the study, a new series of derivatives was designed to explore the influence of different terminal amine groups on the inhibitory activity against electric eel acetylcholinesterase (eeAChE) and equine serum butyrylcholinesterase (eqBuChE), using Ellman’s spectrophotometric method. Selected derivatives were further evaluated to determine their inhibitory activities towards human acetylcholinesterase (hAChE), due to their strong and selective inhibition of both eeAChE and MAO-B. The inhibitory potential of the tested compounds against MAOs was assessed directly on human MAO enzymes using a fluorescence-based Amplex Red method. Donepezil, along with two MAO inhibitors-rasagiline and iproniazid were used as positive controls to benchmark the inhibitory potency of all synthesized compounds. Derivatives 4-((3-chloro-4-methyl-2-oxo-2H-chromen-7-yl)oxy)butyl-2-methylpiperidine-1-carbodithioate (**12**) and 4-((3-chloro-4-methyl-2-oxo-2H-chromen-7-yl)oxy)butyl-2,6-dimethylpiperidine-1-carbodithioate (**13**) (Figure 9) exhibited potent inhibition of *h*MAO-B (IC_50_ = 0.876 ± 0.036 μM and 0.101 ± 0.024 μM, respectively) ascompared to rasagiline IC_50_ = 0.138 ± 0.004 μM).

Compound **13** also exhibited well-balanced inhibitory activity against eeAChE (IC_50_ = 0.044 ± 0.002 μM). Kinetic and molecular docking studies indicated that it acts as a dual-site inhibitor of AChE and a competitive antagonist of MAO-B. Moreover, the compound demonstrated effective blood–brain barrier permeability (Pe = 5.75 ± 0.11 × 10^−6^ cm/s), showed no cytotoxicity toward SH-SY5Y neuronal cells, and significantly improved cognitive deficits in a scopolamine-induced Alzheimer’s disease mouse model [38,39,40,41,42,43,44,45].

**Figure 10:**
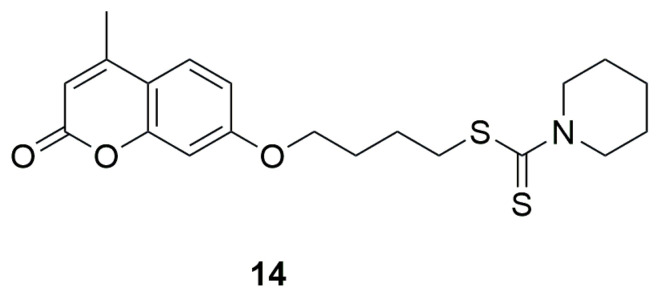
Structure of coumarin derivative 4-((4-methyl-2-oxo-2H-chromen-7-yl)oxy)butylpiperidine-1-carbodithioate (**14**).

In 2018, Jiang and colleagues developed a new series of multifunctional coumarin–dithiocarbamate hybrids using 7-hydroxy-4-methyl-2H-chromen-2-one, different secondary amines and carbon disulfide as potential therapeutic agents for Alzheimer’s disease [46]. Biological evaluations demonstrated that many of the synthesized compounds strongly inhibited acetylcholinesterase (AChE) and showed high selectivity over butyrylcholinesterase (BChE). Among them, 4-((4-Methyl-2-oxo-2H-chromen-7-yl)oxy)butyl piperidine-1-carbodithioate (**14**) (Figure 10) emerged as the most potent AChE inhibitor, with an IC_50_ value of 0.027 ± 0.002 μM. At a concentration of 25 μM, it also inhibited Aβ aggregation by 40.19 ± 2.39%. Additionally, compound **14** exhibited metal-chelating properties, favorable blood–brain barrier permeability, and low cytotoxicity in SH-SY5Y human neuroblastoma cells. In vivo, it showed no acute toxicity at doses up to 1000 mg/kg and significantly improved cognitive performance in a scopolamine-induced AD mouse model—demonstrating AChE inhibition 1.5 times stronger than donepezil [38,39,40,41,42,43,44,45].

### 2.3. Coumarin–Dithiocarbamate Derivatives with α-Glucosidase Inhibitory Effects

Type 2 diabetes mellitus is a chronic metabolic disorder in which cells are resistant to insulin, resulting in persistent hyperglycemia. One of main contributors to postprandial blood sugar spikes in individuals with type 2 diabetes is rapid breakdown of carbohydrates in the digestive system, which is facilitated by enzymes such as alpha-glucosidase, which catalyzes converting complex polysaccharides into absorbable glucose. Given this role, inhibiting alpha-glucosidase activity is considered an effective strategy for the treatment of diabetes mellitus.

**Figure 11:**
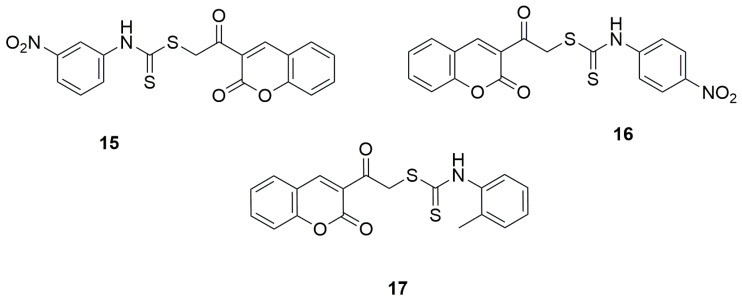
Coumarin derivatives synthesized by Elahabaadi’s group [47].

In 2021, two independent studies were conducted, where series of novel coumarin–dithiocarbamate derivatives were designed, synthesized and tested for alpha-glucosidase inhibitory activities by preparing mixture of alpha-glucosidase (Saccharomyces cerevisiae, EC3.2.1.20), potassium phosphate buffer and different concentrations of synthesized compounds in 96-well plate, incubating for 10 min in 37 °C, adding *p*-nitrophenyl glucopyranoside and incubating in 37 °C for 20 min. DMSO (10% final concentration) was used as negative control and acarbose was used as positive control. Finally, absorbance was detected at 405 nm and IC_50_ values were calculated. Among the group of 3-(2-[(phenylcarbamothioyl)sulfanyl]acetyl)-2H-chromen-2-one derivatives synthesized by Elahbaadi and co-workers, the most potent inhibitory activity was exhibited by 2-oxo-2-(2-oxo-2H-chromen-3-yl)ethyl(3-nitrophenyl)carbamodithioate (**15**) and 2-oxo-2-(2-oxo-2H-chromen-3-yl)ethyl(4-nitrophenyl)carbamodithioate (**16**)**,** with IC_50_ values of 101.6 ± 4.7 µM and 85.0 ± 4.0 µM, respectively [47] (Figure 11). In comparison, acarbose showed an IC_50_ of 750.0 ± 9.0 µM in the same study. However, it should be emphasized that all the evaluated compounds exhibited markedly stronger inhibitory effects than the reference drug acarbose. However, compounds containing electron-withdrawing substitutions on the aryl group, such as nitro groups or halogens, showed the highest activity. The activity slightly decreased for derivatives with an electron-donating substituents, like a methyl group in the ortho position of the phenyl moiety, IC_50_ = 560.6 ± 8.6 for 2-oxo-2-(2-oxo-2H-chromen-3-yl)ethyl-*o*-tolylcarbamodithioate (**17**) [47] (Figure 11).

In contrast to the previously discussed study, where coumarin derivatives substituted with a dithiocarbamate group at the C-3 position of the coumarin ring were synthesized, the group led by Mollazadeh and collaborators investigated derivatives substituted at the C-4 position and additionally containing a methoxy group at the C-7 position [48].

**Figure 12:**
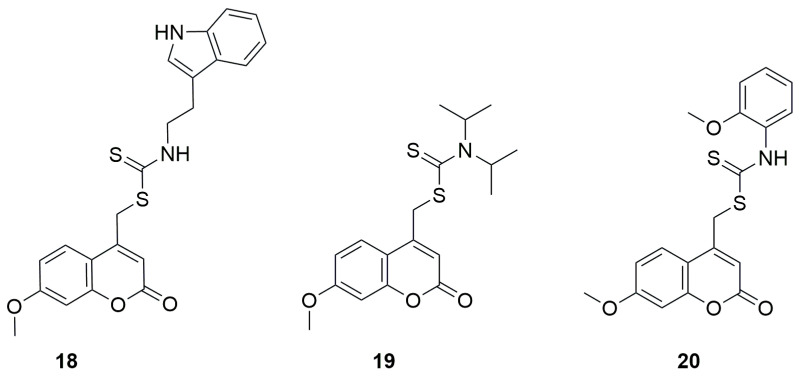
Structure of coumarin derivative (7-methoxy-2-oxo-2H-chromen-4-yl)methyl-2-(1H-indol-3-yl)ethylcarbamodithioate (**18**), (7-methoxy-2-oxo-2H-chromen-4-yl)methyldiisopropylcarbamodithioate (**19**) and (7-methoxy-2-oxo-2H-chromen-4-yl)methyl 2-methoxyphenylcarbamodithioate (**20**).

Here as well, the newly synthesized derivatives exhibited better inhibitory potential than acarbose, with IC_50_ values ranging from 85.0 to 566.6 μM. The compounds showing the strongest activity were **18**, an indoleethanamine derivative from the secondary amine group, and **19**, a diisopropylamine derivative from the tertiary amine group, with IC_50_ values of 85.0 ± 4.0 μM and 100.6 ± 4.7 μM, respectively (Figure 12). It was found that the aniline derivative (**20**) also exhibited good inhibitory activity against α-glucosidase, but its analogs containing methylene or ethylene groups between the NH unit and the aromatic moiety showed stronger inhibitory activity than aniline derivative **20**. On the other hand, elimination of the aromatic group from the amine portion resulted in a decrease in inhibitory activity. Replacement of the diisopropylamine group with piperidine or methylpiperazine led to a significant decrease in α-glucosidase inhibitory activity. In contrast, the introduction of aromatic groups such as phenyl, 4-methylphenyl, 2-methoxyphenyl, 4-methoxyphenyl or 3-fluorophenyl in place of the methyl group on the piperazine ring improved the inhibitory activity.

### 2.4. Coumarin–Dithiocarbamate Derivatives with Antimicrobial, Antifungal, and Antiparasitic Activity

Bacterial and fungal infections are significant contributors to various health disorders. For many years, research has been ongoing to identify effective antimicrobial agents capable of combating these conditions. So far, several studies have been published demonstrating the potential of dithiocarbamate derivatives of coumarins in this field.

**Figure 13:**
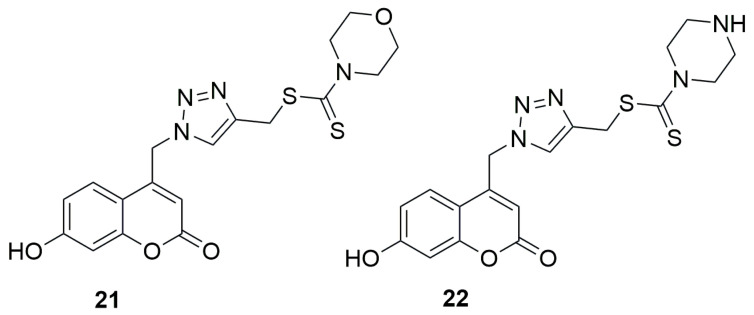
Structures of {1-[(7-hydroxy-2H-chromen-2-one-4-yl)methyl]-1H-1,2,3-triazol-4-yl}methylthio)carbonothioyl)morpholine (**21**) and {1-[(7-hydroxy-2H-chromen-2-one-4-yl)methyl]-1H-1,2,3-triazol-4-yl}methylthio)carbonothioyl)piperazine (**22**).

In 2016, Krajlevic et al. investigated the in vitro antibacterial activity of novel 1,2,3-triazole–coumarin conjugates against Gram-positive bacteria, including *Staphylococcus aureus* (ATCC 25923), *Enterococcus faecalis*, and vancomycin-resistant *Enterococcus faecium* (VRE), as well as Gram-negative bacteria such as *Pseudomonas aeruginosa* (ATCC 27853), *Escherichia coli* (ATCC 25925), *Acinetobacter baumannii* (ATCC 19606), and extended-spectrum β-lactamase (ESBL)-producing *Klebsiella pneumonia* [49]. The results were compared with those obtained for the well-known antibiotics ceftazidime (CAZ) and ciprofloxacin (CIP). Minimum inhibitory concentrations (MICs) were determined to assess the antibacterial activity of the synthesized compounds. Coumarin-1,2,3-triazole hybrids containing 4-(methylthiocarbonothioyl)morpholine (**21**) and 4-(methylthio carbonothioyl)piperazine (**22**) moieties demonstrated notable antibacterial potency against *E. faecalis*, with MIC values of 32 µg/mL and 16 µg/mL, respectively (Figure 13). The reference compound CAZ was inactive in this case, with an MIC of 256 µg/mL, whereas CIP exhibited an MIC value of 0.5 µg/mL.

**Figure 14:**
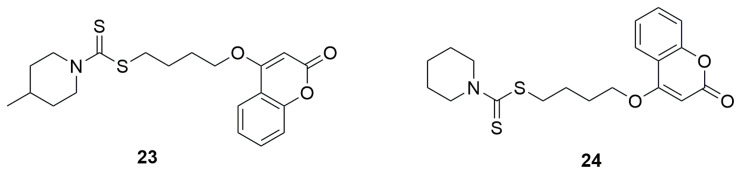
Structures of 4-(2-oxo-2H-chromen-4-yloxy)butyl-4-methylpiperidine-1-carbodithioate (**23**) and 4-(2-oxo-2H-chromen-4-yloxy)butyl piperidine-1-carbodithioate (**24**).

In another study, a series of coumarin–dithiocarbamate hybrid compounds was identified as promising candidates for antimicrobial agents [50]. Among them, 4-(2-oxo-2H-chromen-4-yloxy)butyl-4-methylpiperidine-1-carbodithioate (**23**) exhibited notably strong antimicrobial activity against all tested bacterial and fungal strains with an MIC of 0.5 µg/mL against *S. aureus*, 1 µg/mL against *B. subtilis*, and 2 µg/mL against *E. coli*, and *P. aeruginosa* (Figure 14). Replacing the methylpiperidine fragment with a piperidine moiety also yielded good results. 4-(2-oxo-2H-chromen-4-yloxy)butyl piperidine-1-carbodithioate (**24**) demonstrated good activity, showing an MIC of 1 µg/mL for *S. aureus*, *B. subtilis*, and *P. aeruginosa*, as well as 1 µg/mL for *E. coli*. In both cases, a four-carbon linker was used between the coumarin and dithiocarbamate moieties.

Similarly, compounds containing a butyl linker and pyrrolidine or methylpiperazine fragments also exhibited good activity, with MIC values in the range of 2–8 µg/mL. Ciprofloxacin, used as the reference compound, exhibited an MIC = 2 µg/mL in each case. On the other hand, replacing the butyl chain with a two-carbon fragment drastically reduced the antibacterial activity.

The antifungal activity was evaluated against fungal strains such as *A. flavus*, *T. harzianum*, *P. chrysogenum*, and *C. albicans*, and Fluconazole was used as the standard drug. Similar to the antibacterial studies, compounds **23** and **24** showed the most potent activity. Derivative **23** exhibited a MIC value of 0.5 µg/mL against *A. flavus*, *T. harzianum*, and *P. chrysogenum*, and 1 µg/mL against *C. albicans*. Compound 24 showed a MIC of 1 µg/mL against *A. flavus*, *T. harzianum*, and *C. albicans*, and 0.5 µg/mL against *P. chrysogenum.* Fluconazole, used as the reference compound, exhibited an MIC = 2 µg/mL in each case.

**Figure 15:**
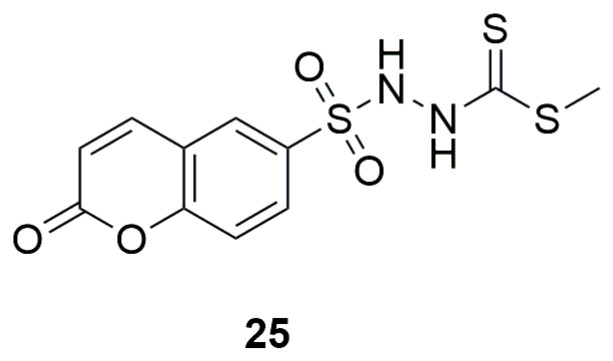
Structure of methyl 2-[(coumarin-6-yl)sulfonyl]hydrazine-1-carbodithioate (**25**).

In recent studies conducted in 2025, a new small molecule, methyl 2-[(coumarin-6-yl)sulfonyl]hydrazine-1-carbodithioate (**25**), was investigated for its antimicrobial activity and ability to inhibit biofilm formation against various test microorganisms (Figure 15) [51]. The results of the antimicrobial assays revealed that compound **25** exhibited notable antibacterial activity, with MIC values of 78.43 ± 1.928 μg/mL against *S. aureus* and 158.67 ± 2.137 μg/mL against *E. coli*. It showed even greater potency against *C. albicans* and *A. niger*, with MIC values of 20.27 ± 2.359 μg/mL and 9.71 ± 0.702 μg/mL, respectively, surpassing the reference drug nystatin. Evaluation of anti-biofilm activity demonstrated that methyl 2-[(coumarin-6-yl)sulfonyl]hydrazine-1-carbodithioate effectively inhibited biofilm formation by *S. aureus* (MIC: 55.91 ± 1.642 μg/mL, 1.5-fold higher than the reference) and *C. albicans* (MIC: 78.705 ± 2.048 μg/mL, 4-fold higher than the reference).

**Figure 16:**
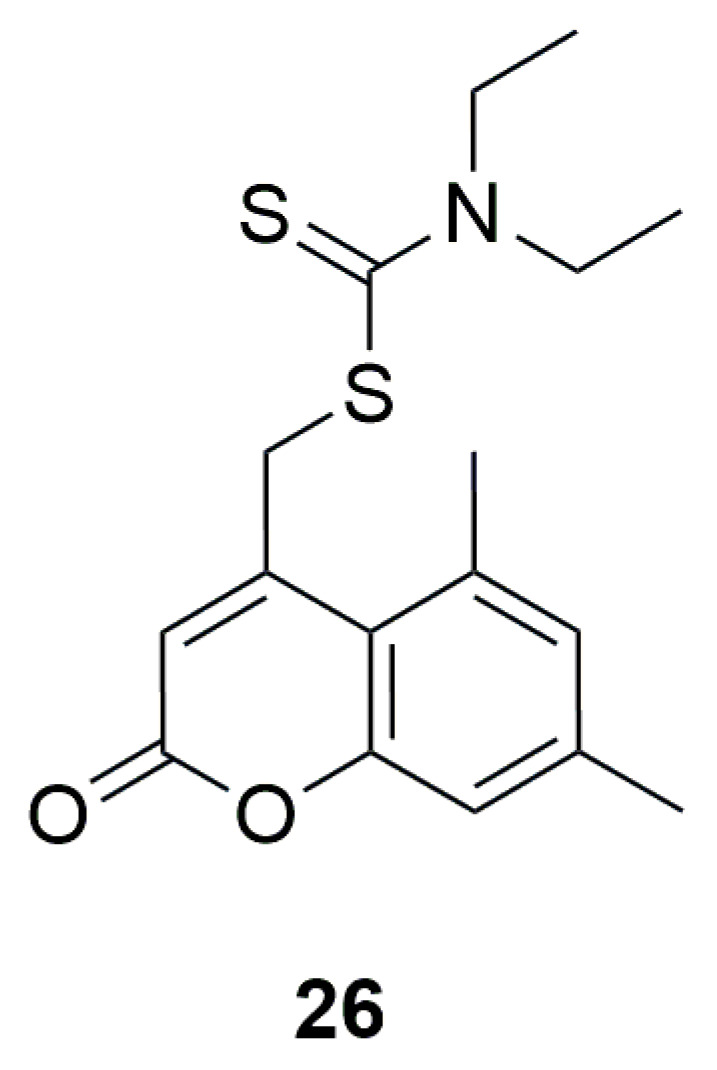
Structure of (5,7-dimethyl-2-oxo-2H-chromen-4-yl)-methyl diethyl dithiocarbamate (**26**).

An interesting point is the theoretical research conducted in 2018 on (5,7-dimethyl-2-oxo-2H-chromen-4-yl)-methyl diethyldithiocarbamate (Figure 16). Molecular docking studies, performed using Schrodinger software to assess potential antimicrobial activity, indicated that this compound possesses promising antifungal properties due to the strong predicted interaction and good ligand–protein fit with antifungal properties (5HS1). However, no in vitro studies were carried out to confirm these findings [52].

**Figure 17:**
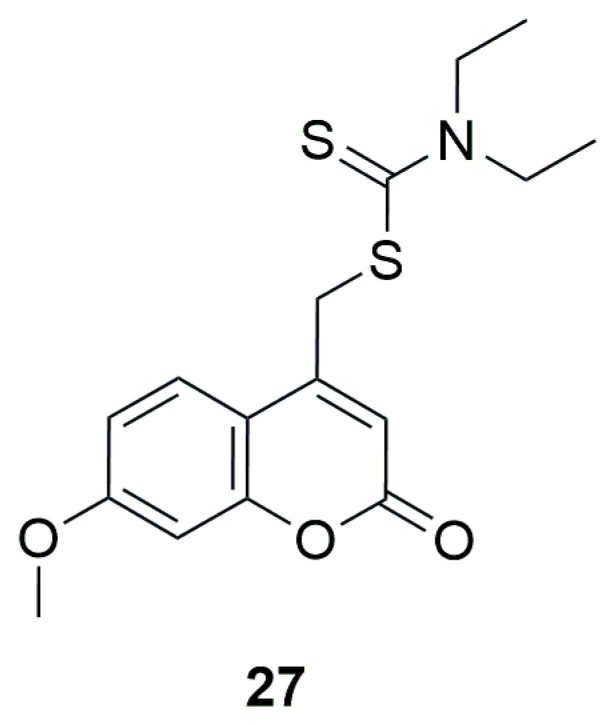
Structure of ((7-methoxy-2-oxochromen-4-yl)methyl)-*N*,*N*-diethyldithiocarbamate (**27**).

In 2022, it was demonstrated that dithiocarbamate analogs exhibit promising antischistosomal activity. In their work, Rannar et al. studied over a hundred compounds with this structure, including one coumarin derivative [53]. ((7-Methoxy-2-oxochromen-4-yl)methyl)-*N*,*N*-diethyldithiocarbamate (**27**) was active down to 5 μM in terms of pairing instability, unsucked schistosomes, inhibition of egg production, and 10 μM in terms of reduction of motility (Figure 17).

## 3. Conclusions

As demonstrated in this study, coumarin-based dithiocarbamate derivatives exhibit a wide range of biological activities. We hope that our summary clearly shows that such hybrid systems display promising α-glucosidase inhibitory effects, antimicrobial activity, an impact on the central nervous system, and promising cytotoxic activity. Due to their potential broad applications, these compounds can be considered as scaffolds for the development of new lead structures and, consequently, as future drug candidates for combating serious global diseases. It is also worth noting that this topic remains relatively underexplored in the scientific literature. Research on biologically active coumarin–dithiocarbamate hybrids began to emerge only after 2013, and the number of studies is still limited—which presents a strong rationale for further, exciting research in this field.
